# Targeting inerleukin-6 for renoprotection

**DOI:** 10.3389/fimmu.2024.1502299

**Published:** 2024-12-11

**Authors:** Ekaterina O. Gubernatorova, Mikhail Y. Samsonov, Marina S. Drutskaya, Svetlana Lebedeva, Darya Bukhanova, Maria Materenchuk, Kerim Mutig

**Affiliations:** ^1^ Engelhardt Institute of Molecular Biology, Russian Academy of Sciences, Moscow, Russia; ^2^ Center for Precision Genome Editing and Genetic Technologies for Biomedicine, Engelhardt Institute of Molecular Biology, Russian Academy of Sciences, Moscow, Russia; ^3^ R-Pharm JSC, Medical Department, Moscow, Russia; ^4^ Sirius University of Science and Technology, Federal Territory Sirius, Krasnodarsky Krai, Russia; ^5^ Department of Pharmacology, Institute of Pharmacy, I.M. Sechenov First Moscow State Medical University, Moscow, Russia; ^6^ Department of Medical Elementology, Peoples’ Friendship University of Russia (RUDN University), Moscow, Russia

**Keywords:** acute kidney injury, chronic kidney disease, inflammation, anti-cytokine therapy, kidney transplantation, trans-signaling, IL-6

## Abstract

Sterile inflammation has been increasingly recognized as a hallmark of non-infectious kidney diseases. Induction of pro-inflammatory cytokines in injured kidney tissue promotes infiltration of immune cells serving to clear cell debris and facilitate tissue repair. However, excessive or prolonged inflammatory response has been associated with immune-mediated tissue damage, nephron loss, and development of renal fibrosis. Interleukin 6 (IL-6) is a cytokine with pleiotropic effects including a major role in inflammation. IL-6 signals either via membrane-bound (classic signaling) or soluble receptor forms (trans-signaling) thus affecting distinct cell types and eliciting various metabolic, cytoprotective, or pro-inflammatory reactions. Antibodies neutralizing IL-6 or its receptor have been developed for therapy of autoimmune and chronic non-renal inflammatory diseases. Small molecule inhibitors of Janus kinases acting downstream of the IL-6 receptor, as well as recombinant soluble glycoprotein 130 variants suppressing the IL-6 trans-signaling add to the available therapeutic options. Animal data and accumulating clinical experience strongly suggest that suppression of IL-6 signaling pathways bears therapeutic potential in acute and chronic kidney diseases. The present work analyses the renoprotective potential of clinically relevant IL-6 signaling inhibitors in acute kidney injury, chronic kidney disease, and kidney transplantation with focus on current achievements and future prospects.

## Graphical Abstract

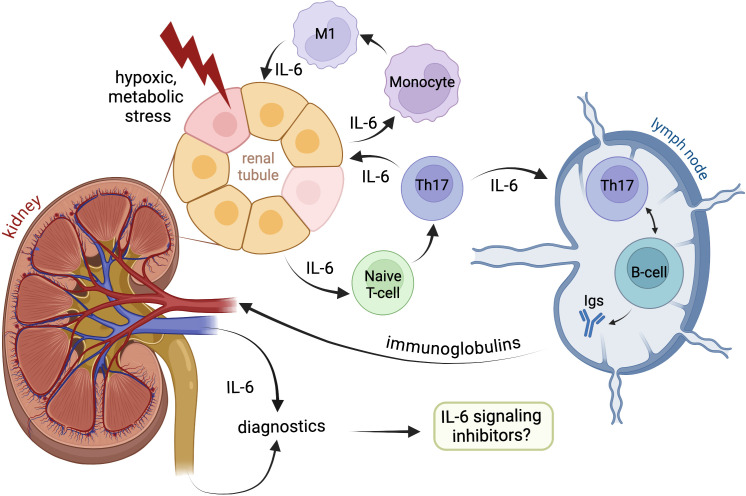

## Principal interleukin-6 signaling

Interleukin-6 (IL-6) is a pivotal cytokine with pleiotropic cell biologic and physiologic functions ranging from immunomodulatory to metabolic effects. Structurally, IL-6 belongs to the four-helical cytokine family and shares homology with IL-11, ciliary neurotrophic factor (CNTF), leukemia inhibitory factor (LIF), oncostatin M (OSM), cardiotrophin 1 (CT-1), cardiotrophin-like cytokine (CLC), IL-27, and IL-31, together referred to as the IL-6 cytokine family (1, 2). The IL-6 family members exert partially overlapping, as well as distinct effects with IL-6 being a major player in mediating inflammation. The cytokine promotes T-cell and B-cell immune responses via enlarging the pro-inflammatory T-helper 17 (Th17) and M1 macrophage subsets, boosting the antibody production by B-cells, and suppressing the anti-inflammatory regulatory T cells (Treg) and M2 macrophages (3–5). Along with the aggressive pro-inflammatory effect spectrum in immune cells, IL-6 exerts adaptive, cytoprotective, and proliferative effects in non-myeloid cells contributing to tissue repair but also provoking malignant cell growth (2, 6–8). Dysregulation of IL-6 signaling has been implicated in autoimmunity and immune-mediated organ damage during sterile inflammation (9).

**Figure 1:**
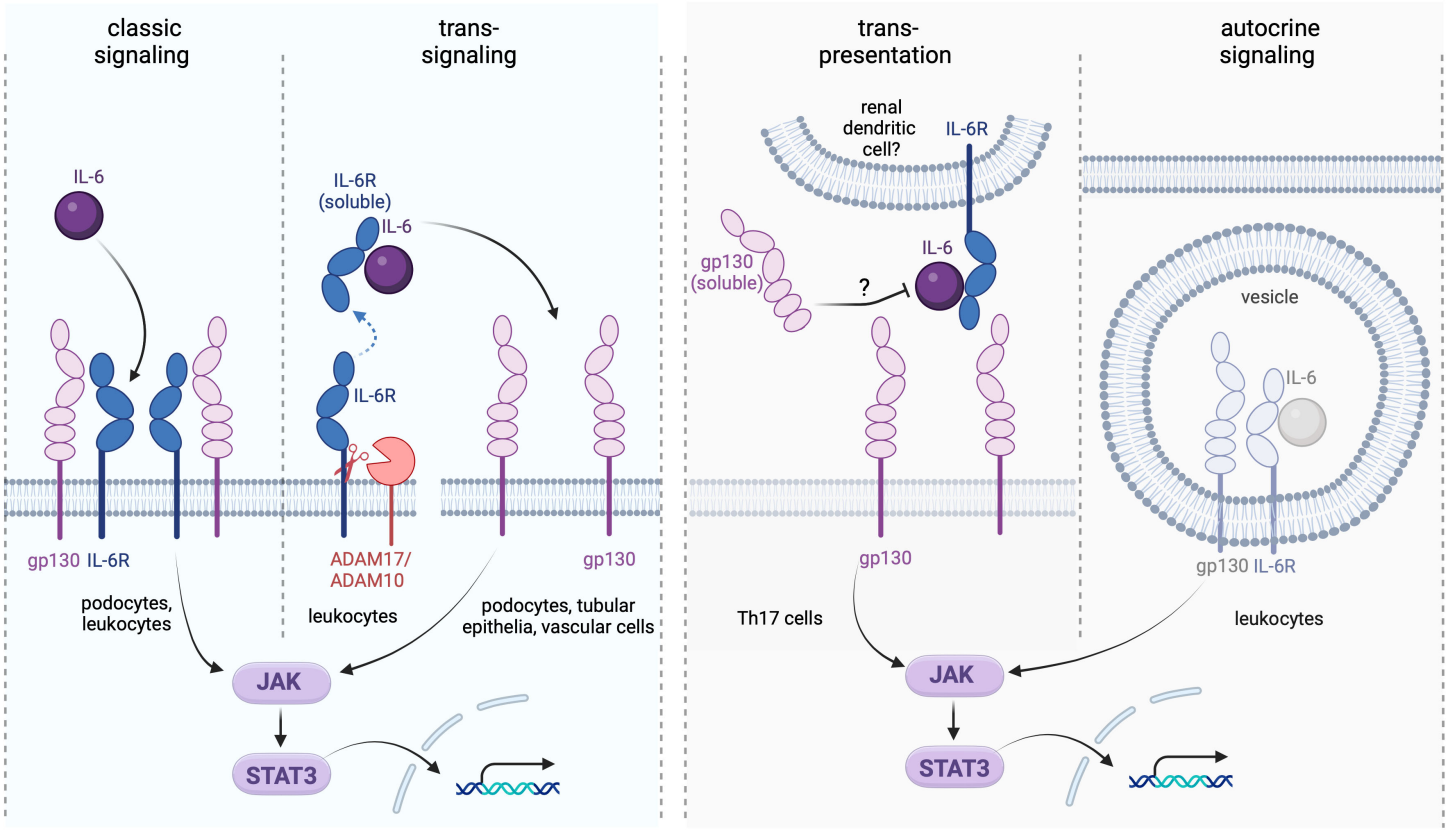
Interleukin-6 (IL-6) signaling modes and their renal targets. The classic signaling takes place in podocytes expressing the membrane-bound IL-6 receptor (IL-6R) and may help maintaining the integrity of the glomerular filtration barrier. The trans-signaling recruits the soluble IL-6R (sIL-6R) generated by proteolytic cleavage involving the proteases ADAM17 and ADAM10. This type of signaling affects any kidney cell expressing gp130 independently on the presence of mIL-6R. The trans-presentation (cluster) mode may involve renal dendritic cells in local interactions with proinflammatory Th17 cells, in analogy to the reported trans-presentation setting in the brain tissue (16). The autocrine IL-6 signaling may occur intracellularly in cell types expressing IL-6, IL-6R, and gp130 simultaneously and likely contributes to pro-inflammatory immune cell reactions; although its role in the kidney requires further elucidation (15). While effects of the classic and trans-signaling pathways in the kidney have been well documented, the relevance of the trans-presentation and autocrine signaling modes in the renal physiology and pathophysiology remains speculative.

IL-6 can be produced by T and B lymphocytes, fibroblasts, monocytes, keratinocytes, mesangial cells, endothelial cells, and subsets of epithelial cells (1). IL-6 expression in intact tissues is typically low but strongly induced during inflammation. Effects of the cytokine are mediated by three distinct signaling pathways referred to as the classic, the trans, and the trans-presentation or cluster modes (10). In the classic signaling, IL-6 first builds a dimer with the glycoprotein 80 (gp80) residing in the plasma membrane and constituting the membrane-bound IL-6 receptor alpha subunit (mIL-6R). The ensuing recruitment of the membrane-bound gp130 (mgp130) acting as the beta IL-6R subunit, followed by the assembly of two IL-6/IL-6R/mgp130 trimeric complexes into a functional hexamer initiate the signal transduction (11, 12). In view of the ubiquitous gp130 expression pattern, the classic signaling is restricted to the cell types possessing mIL-6R. This signaling mode has been primarily implicated in intact cell metabolism and functionality, whereas the pro-inflammatory cytokine effects are predominantly mediated by the trans-signaling (10, 13). The latter is enabled by the circulating soluble IL-6R form (sIL-6R) interacting with IL-6 followed by binding of the resulting IL-6/sIL-6R dimer with mgp130. Therefore, the trans-signaling mode does not depend on mIL-6R and exerts broad effects on all mgp130 expressing cells. The sIL-6R form originates from proteolytic cleavage of mIL-6R provided by metalloproteinases ADAM17 and ADAM10. Alternative IL-6R splicing may contribute to a minor extent to the sIL-6R generation in human (14). Shedding of IL-6R occurs mainly in neutrophils, monocytes, T helper cells, and hepatocytes and is enhanced during inflammation prompting initiation of the trans-signaling. Notably, a naturally occurring soluble gp130 variant (sgp130) acts as endogenous inhibitor of the trans-signaling by buffering the circulating IL-6/sIL-6R complexes and downregulating pro-inflammatory responses in normal conditions (14). In addition to the trans-signaling, sgp130 blunts the trans-presentation (cluster signaling), which is a paracrine interaction requiring a close spatial contact between a mgp130-expressing cell and a cell presenting preformed IL-6/mIL-6R complexes to the former (15). Specifically, the trans-presentation has been implicated in acquisition of the Th17 phenotype during neuroinflammation and development of cytotoxic CD8(+) T cells in the liver (16, 17). It is tempting to speculate that renal dendritic cells may mediate such effects on the infiltrating immune cells as well. Finally, an autocrine intracellular IL-6 signaling inaccessible to sgp130 has been identified *ex vivo* but its contribution to inflammatory processes *in vivo* requires further investigation (15). All IL-6 signaling modes share the downstream signal transducing pathway mediated by Janus Kinases (JAK) providing activating phosphorylation to the Signal Transducer and Activator of Transcription 3 (STAT3) (1). The cellular response to IL-6 is further modulated by Suppressors of Cytokine Signaling (SOCs) that integrate distinct effects of pro- vs. anti-inflammatory cytokines downstream of STAT3 (18). **Figure 1** depicts the IL-6 signaling pathways with a focus on the kidney.

## Clinically relevant inhibitors of IL-6 signaling

**Figure 2:**
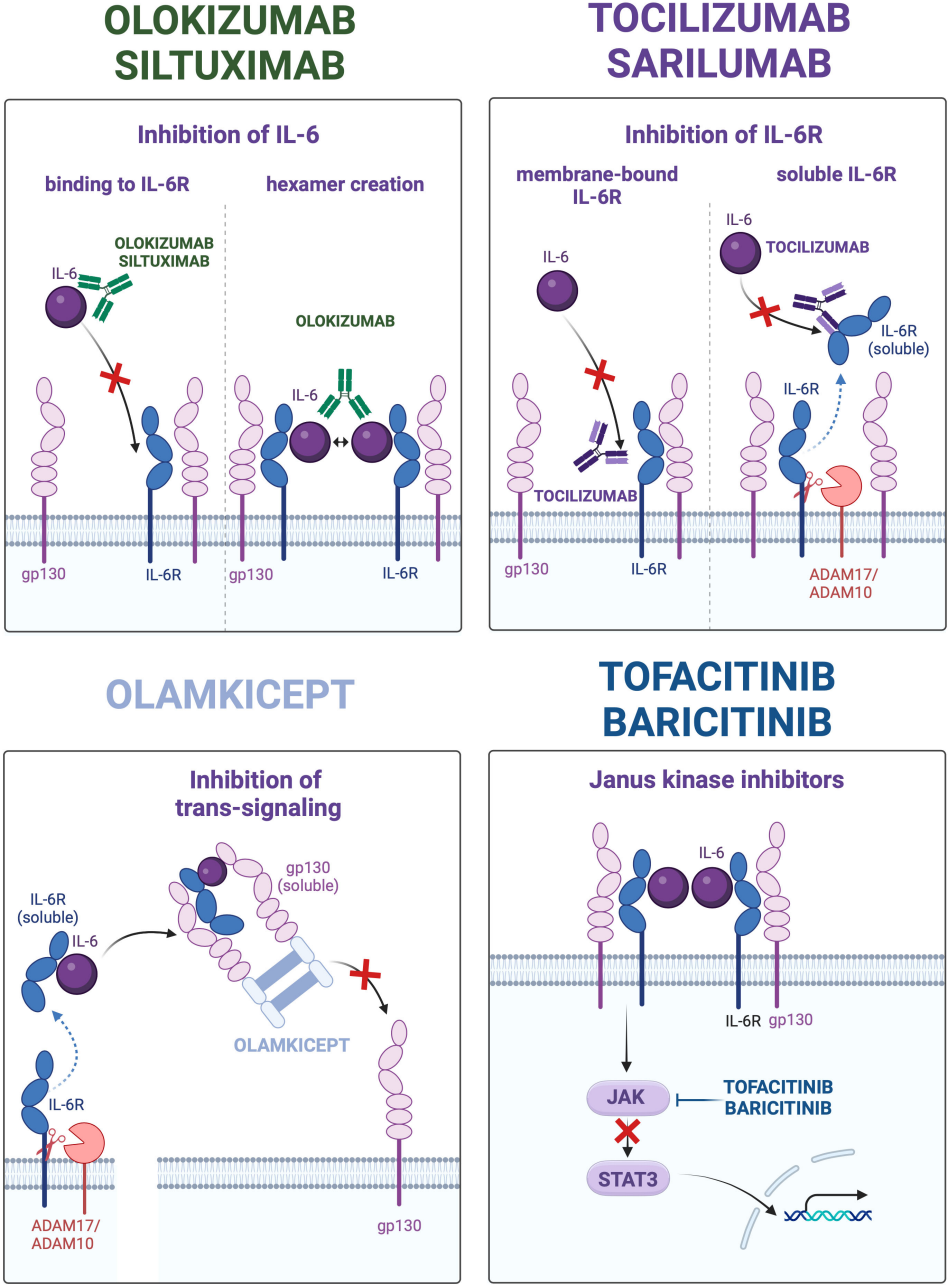
Distinct types of interleukin 6 (IL-6) signaling inhibition in therapeutic approaches. The four panels depict mechanisms of IL-6 signaling suppression utilized by monoclonal antibodies to IL-6 (left upper panel), monoclonal antibodies to IL-6 receptor (IL-6R; right upper panel), modified soluble glycoprotein 130 (gp130) variant (olamkicept; left lower panel), and small molecule inhibitors of Janus Kinases (JAK) – Signal Transducer and Activator of Transcription 3 (STAT3). The representative clinically relevant drugs are mentioned at respective panels.

IL-6 signaling suppressing agents that have been approved or are in development for clinical use can be classified according to their modes of action. *Monoclonal antibodies targeting IL-6* include clazakizumab, olokizumab, siltuximab and sirukumab. These antibodies suppress the classic and the trans-signaling pathways, whereas the trans-presentation (cluster) signaling remains active due to preforming of the IL-6/IL-6R complex inside the presenting cells (16). Only olokizumab is potentially able to suppress the cluster signaling along with the classic and trans-pathways due to specificity of the targeted IL-6 epitope (site III) prohibiting formation of functional hexamers from the two neighboring IL-6/IL-6R/gp130 complexes. To this end, olokizumab may bind with and inactivate the IL-6/IL-6R complex at the surface of the presenting cell thereby inhibiting the trans-presentation pathway (11, 19). Since the trans-presentation has been shown to promote development of Th17 and cytotoxic T cells (16), its suppression may add to the anti-inflammatory and immunosuppressive effects of olokizumab. Furthermore, occupation of mgp130 with inactive olokizumab/IL-6/IL-6R complexes may to a certain extent attenuate effects of other IL-6 family cytokines signaling via mgp130. *Monoclonal antibodies to IL-6R* such as tocilizumab or sarilumab bind to both membrane-bound and soluble receptor form, thereby suppressing the classic and the trans-signaling pathways. In contrast to the former two groups, a *modified soluble gp130 variant* (olamkicept) binds with the circulating IL-6/sIL-6R complex thereby selectively inactivating the IL-6 trans-signaling (10). Since at least IL-11 signals via soluble receptors and gp130 as well, olamkicept suppresses the trans-signaling of both cytokines. Next generations of IL-6 trans-signaling inhibitors with improved selectivity for IL-6 over IL-11 and higher bioavailability have been developed and are in the preclinical testing (10). Finally, a number of small molecules acting downstream of IL-6R such as *JAK inhibitors* have been identified and clinically approved including abrocitinib, baricitinib, delgocitinib, fedratinib, filgotinib, oclacitinib, pacritinib, peficitinib, ruxolitinib, tofacitinib, and upadacitinib (20). **Figure 2** presents distinct mechanisms of IL-6 signaling targeted by the aforementioned agents.

## Interleukin 6 in renal (patho)physiology

### Renal expression and function of the IL-6 signaling components

Among kidney epithelial and vascular cells, expression of mIL-6R takes place only in podocytes and glomerular mesangial cells conferring them responsiveness to the classic IL-6 signaling mode (21, 22). No IL-6R shedding was detected in cultured human glomerular epithelial cells suggesting that podocytes or glomerular mesangial cells provide no significant contribution to circulating or urinary sIL-6R levels *in vivo* (21). Further experimental studies in cell culture and animal models demonstrated the IL-6-induced STAT3 activation in podocytes corroborating the functionality of mIL-6R in this cell type (21–24). The classic IL-6 signaling promotes proliferation of glomerular mesangial cells and mediates podocyte hypertrophy (25, 26). Therefore, IL-6 may contribute to the integrity of the glomerular filtration barrier upon challenge and participate in glomerular hypertrophy and matrix expansion during pathophysiological hyperfiltration. Consistent with the latter assumption, blocking the JAK-STAT3 signaling downstream of IL-6 has been associated with protective effects in various experimental models of glomerular injury and kidney diseases (13, 22–24, 27). The cytokine is obviously dispensable for the intact embryonic kidney development, since pharmacologic IL-6 blockade or genetic IL-6 knockout produced no overt renal phenotype in mice (28). In contrast, embryonic overexposure to IL-6 during pregnancy due to maternal obesity has been implicated in reduction of kidney to body weight ratio in newborns (29). Likewise, administration of IL-6 to mice during gestation resulted in decreased kidney weight and suppression of nephrogenic zone, but facilitated glomerular maturation in pups suggesting that dysregulation of IL-6 signaling during embryonic kidney development may predispose to adult kidney pathology (29). Notably, enhanced circulating IL-6 levels frequently correlate with albuminuria, renal hypertrophy, tubular injury, and intestinal fibrosis suggesting that exaggerated stimulation by IL-6 may deplete the functional podocyte reserve and promote glomerular damage (30).

In contrast to mIL-6R that is expressed solely in glomeruli, production of IL-6 can take place virtually in all kidney epithelial and endothelial cells to a variable extent (21, 31–34). While immune cells are mainly responsible for circulating IL-6 levels under homeostatic conditions, the kidneys become a major cytokine source in pathophysiological situations associated with acute kidney injury (AKI) or chronic kidney disease (CKD) (35). Both injured kidney tissue and infiltrating immune cells significantly contribute to elevated local and systemic IL-6 levels in kidney disease (36). Experimental studies implicate IL-6 in mediating tubular damage since selective suppression of the downstream JAK-STAT3 signaling reduced acute kidney injury and alleviated diabetic kidney fibrosis in mice (37, 38). Furthermore, excessive IL-6 signaling may promote the epithelial-mesenchymal transition (EMT) thus enhancing risk of the clear-cell renal cell carcinoma (ccRCC) (39). These pathophysiological effects are predominantly mediated by the trans-signaling. From the physiologic point of view, local production of the cytokine may be involved in functional and structural adaptations of glomerular and tubular epithelia to perturbations of water-, electrolyte- or protein load. The available data on distribution and functions of IL-6 and IL-6R in the kidney are summarized in the **Table 1**.

**Table 1:** Expression, physiologic functions, and pathophysiologic implications of interleukin-6 signaling in kidney cells.

Cell type	IL-6	mIL-6R	Physiology	Pathophysiology	Citations
Podocytes	+	+	Functional and structural adaptations	Hypertrophy, overload stress	(21–24)
Mesangial cells	+	–	Proliferation	Matrix expansion	(31–33)
Tubular epithelia	+	–	Functional and structural adaptations	Sterile inflammation. tubulo-interstitial damage, fibrosis	(36, 37, 136)
Endothelial cells	+	–	Cytokine and chemokine release, coagulation	Vascular leakage, oxidative stress, abnormal coagulation, tissue damage	(34, 202, 203)
Fibroblasts	+	–	Proliferation	Proliferation, fibroblast-to-mesenchymal transition, fibrosis	(204)
Dendritic cells	+	–	Innate immunity	Immune-mediated damage	(35, 59)
Macrophages	+	–	Innate immunity	Immune-mediated damage	(58, 205)

+, readily detectable expression levels.-, undetectable expression.

Expression of gp130 appears to be ubiquitous in the kidney since the classic or trans IL-6 signaling modes were readily detected in renal cell cultures or animal models of kidney diseases (35). Finally, SOCs interfering with IL-6-induced STAT3 activation are broadly expressed across the kidney epithelia as well (27).

### Effects of IL-6 on the kidney function and hemodynamics

Blood filtration is critical to the body homeostasis. To fulfil this task, renal blood perfusion is maintained at constantly high levels (20-25% of cardiac output) by means of autonomous regulation and systemic feedback (40). Experiments in IL-6 knockout mice suggested only minor effects of the cytokine on the intact renal blood flow and glomerular hemodynamics (41). Nevertheless, hormones regulating renal hemodynamics and function such as angiotensin II (AngII) or vasopressin induce changes in IL-6 expression suggesting that the cytokine may mediate some endocrine signaling events (42, 43). Kidney disorders frequently induce or aggravate hypertension and vice versa (44). IL-6 levels have been associated with elevated blood pressure in humans and in experimental animal models (41, 45–49). Increase in physiologic blood pressure in response to physical stress involves IL-6 signaling as well (48). The cytokine may enhance the blood pressure via synergism with the Renin-Angiotensin Aldosterone System (RAAS) since IL-6 deletion blunted the angiotensin II (AngII)-induced hypertension in mice (41, 45, 50). Surprisingly, vasoconstrictive response to AngII was largely preserved in IL-6 knockout mice suggesting non-vascular mechanisms (41). AngII is not only a potent vasoconstrictor but also stimulates tubular salt reabsorption via induction of the aldosterone biosynthesis in the adrenal glands. However, IL-6 knockout mice were not protected against the aldosterone-dependent hypertension (46). Alternatively, pro-hypertensive and pro-fibrotic effects of IL-6 may be mediated by immune cells with pathogenic phenotypes accumulating in the kidney tissue, as suggested by beneficial effects of IL-6 blockade in rats with salt-sensitive hypertension (51). Although details linking IL-6 to the blood pressure remain to be clarified (43, 52, 53), the cytokine has been increasingly recognized as a key player connecting chronic inflammation to hypertension (47, 54, 55).

### IL-6 signaling in the immune-mediated kidney damage

Sterile inflammation is a common pathogenetic feature of acute and chronic non-infectious kidney diseases (56, 57). Kidney injury leads to release of multiple membrane, intracellular, and nuclear compounds from dying cells (urate, ATP, heat-shock proteins, cyclophilins, lipoproteins, histones, etc.) serving as Danger-Associated–Molecular Patterns (DAMPs) and inducing infiltration of immune cells into the kidney tissue independent of primary etiology (58). This process is mediated by dendritic cells and macrophages, which abundantly express Pattern Recognition Receptors (PRR) and release proinflammatory cytokines including IL-6 in response to DAMPs (58–60). The IL-6 induction in immune cells is likely potentiated by paracrine interactions with injured renal cells producing IL-6 as well (36). Therefore, along with protective functions such as clearance of cellular debris and antigen presentation, macrophages contribute to sustained intrarenal exposure to IL-6 via a positive paracrine feedback. Pathophysiologic roles of macrophages in acute and chronic kidney damage are well established (58, 60, 61), whereas the impact of macrophage-derived IL-6 on the development, course, and resolution of kidney disease is less clear. The extent of macrophage invasion and acquisition of distinct macrophage phenotypes in the kidney tissue appear to be critical for the outcome of Acute Kidney Injury (62). Depletion of macrophages before the AKI event has been shown to alleviate kidney damage and promote repair of the kidney tissue in the ischemia-reperfusion AKI mouse model. At the same time, suppression of macrophages several days after the AKI event delayed the tubular repair (62). Suppression of proinflammatory, cytokine-releasing M1 macrophages and promoting their switch to the anti-inflammatory M2 phenotype was shown to reduce inflammation and promote repair of kidney tissue in AKI models (63). Importantly, optimal AKI resolution minimizes the risk of late interstitial fibrosis and transition to CKD. However, translation of experimental data to human disease is complicated by differences in details of immune responses between the human and murine species (64). Examination of macrophage phenotypes in kidney biopsies from patients with acute tubular injury revealed enrichment in both HLA-DR^+^ M1 macrophages and CD163^+^ M2 macrophages (65). Furthermore, increased macrophage density in kidney biopsies correlates with the severity of Chronic Kidney Disease (CKD) and progression to the End-Stage Renal Disease (ESRD) (66). Renal M1 macrophages secrete pro-inflammatory cytokines such as IL-6, which, in turn, switches the differentiation balance of peripheral blood monocytes from dendritic cells to macrophages (35, 67). It is tempting to speculate that M1 macrophage-derived IL-6 is implicated in pathogenesis of AKI and CKD. An enhanced number of the IL-17-producing T helper (Th17) cells along with reduction in the regulatory T cells (Treg) have been further recognized as crucial pathogenetic events in the immune-mediated kidney damage (68, 69). Although TGF-β regulates T-cell-mediated tolerance and immunity through both Treg and Th17 cells, excessive IL-6 availability shifts the CD4-positive T cell differentiation toward Th17 rather than Treg thereby inducing a pathogenic pro-inflammatory imbalance between the two T-cell phenotypes (5).

## Components of IL-6 signaling as kidney disease biomarkers

The serum levels of IL-6 in healthy adults are typically below 5 pg/ml (1-5 pg/ml) being in the lower detection range for the most commercial assays (9). In contrast, circulating levels of sIL-6R (40-75 ng/mL) and sgp130 are much higher (250-400 ng/ml). Calculations based on the molecular weights of IL-6 (~20 kDa), sIL-6R (~55 kDa), and sgp130 (~100 kDa), their circulating levels, and mutual binding affinities imply a limited buffering capacity for suppression of the trans-signaling by sgp130. Thus, initiation of the trans-signaling mainly depends on changes in circulating IL-6 and sIL-6R concentrations (9). Moreover, the balance between circulating IL-6, sIL-6R, and sgp130 may favor either the classic or trans-signaling pathways, as well as permit both signaling modes simultaneously (70). Accordingly, IL-6, sIL-6R, and sgp130 have been receiving growing attention as potential serum or urinary biomarkers in kidney pathology. Several aspects need to be taken into consideration for interpretation of their predictive and prognostic values. The clearance of circulating IL-6 and sIL-6R occurs mainly via their filtration into the urine. Due to the relatively small molecular weights both IL-6 (~20 kDa) and sIL-6R (~55 kDa) pass through the glomerular filtration barrier. In contrast, the IL-6/sIL-6R complex with the molecular weight over 70 kDa, as well as sgp130 (~100 kDa) or the IL-6/sIL-6R/sgp130 complex are mostly retained in the blood assuming the intact glomerular filtration barrier. Therefore, urine of a healthy individual is expected to contain low to moderate levels of IL-6 and sIL-6R. Due to the urine concentration process, the urinary levels of IL-6 and sIL-6R may exceed their plasma concentrations, despite proximal reabsorption of filtered protein. Kidney diseases are frequently associated with impaired glomerular function resulting in proteinuria. Considering the concomitant induction of IL-6 expression in the injured kidney tissue along with impaired proximal protein reabsorption due to tubular damage, significant increases of circulating and especially urinary IL-6 and sIL-6R are typically detected in renal patients even by the reduced GFR. Glomerular damage may, in turn, enhance the urinary IL-6 excretion while reducing its serum levels due to proteinuria. Taken together, urinary IL-6 levels have shown predictive value in adult and pediatric kidney diseases (30, 71). More systematic clinical studies are required to standardize the diagnostic implementation of urinary IL-6 tests in various kidney pathology.

## IL-6 signaling in acute kidney injury

Acute Kidney Injury (AKI) or Acute Renal Failure (ARF) is defined as a sudden loss of kidney function occurring within a short time frame of several hours to several days and leading to insufficient waste filtration from the blood. AKI complicates renal and non-renal diseases in approximately 5% of hospital admissions and 30% of intensive care unit (ICU) admissions being a major cause of morbidity and mortality in hospitalized patients (72). AKI may result from an acute hemodynamical or toxic kidney damage or reflect a decompensation in various chronic kidney disorders. Inadequate AKI resolution may lead to persistent morphological and functional kidney damage with ensuing transition to CKD or ESRD (72). Approximately 40% of patients with renal disease develop acute tubular necrosis manifesting as AKI with ensuing nephron loss. In this light, prediction, early recognition and targeted etiologic and pathogenetic therapy of AKI is of high clinical relevance (72).

Ischemia-reperfusion injury and inflammation of the kidney tissue have been linked with induction of IL-6 expression in AKI patients and experimental animal models (73–75). The damaged kidney is the major source for circulating IL-6 originating both from injured kidney cells and infiltrating immune cells (76). At the same time, the ability of the proximal tubule to reabsorb and metabolize filtrated small proteins including IL-6 is reduced in AKI. Enhanced IL-6 production and impaired renal metabolism of the cytokine lead to substantially elevated urinary IL-6 levels in AKI patients despite the reduced filtration of circulating IL-6 into the urine due to decreased GFR (71). Local IL-6 induction in response to tissue injury followed by reciprocal systemic induction of the cytokine expression in a wide range of white blood cells increase the renal exposure to IL-6 primarily via the trans-signaling mode mediated by sIL-6R (77). Despite numerous reports of enhanced serum or urinary IL-6 levels in association with AKI (71, 78–82), data on simultaneous detection of IL-6 and sIL-6R in serum or urine is rather scarce and mostly derived from kidney transplant recipients experiencing acute graft rejection (83). Synchronized detection of IL-6 and sIL-6R in patients with or at risk of AKI are mandatory for improved understanding of relevant IL-6 signaling modes in this condition. Although IL-6 is primarily viewed as a proinflammatory cytokine promoting renal damage, cell-protective and regenerative effects of IL-6 during kidney injury have been documented as well (77, 84). In fact, boosting the trans IL-6 signaling using a IL-6:sIL-6R fusion protein (hyper-IL-6) exerted marked renoprotective effects in two distinct mouse models of AKI induced either by HgCl_2_ or ischemia-reperfusion (77, 84). These results raise the opportunity of protective rather than pathophysiologic role of IL-6 induction in the injured kidney and challenge the eligibility of pharmacologic IL-6 signaling blockade in AKI patients. Somewhat confusing is the fact that genetic IL-6 deletion protected against HgCl_2_-induced AKI and IgA-mediated kidney damage in mouse models (77, 85). Furthermore, transgenic IL-6 overexpression led to progressive kidney injury resembling the terminal stages of multiple myeloma (myeloma kidney) (86). The aforementioned discrepancies in IL-6 effects may be related with predominant activation of either the classic or the trans-signaling modes, as well as with distinct immunologic status in IL-6 deficient mice vs. wild-type or IL-6 overexpressing mice subjected to AKI. The immune-mediated damage plays a major role in AKI (62). Suppression of the M1 macrophage-dependent acute pro-inflammatory response was beneficial in AKI mouse models (63), whereas the anti-inflammatory M2 macrophages were shown to mediate the kidney repair in the late AKI phase (62). Likewise, increases in circulating and renal Th17 cells have been associated with AKI in humans and rodent models (87), whereas Tregs exert renoprotective effects (88). Since IL-6 promotes the maturation of M1 macrophages and Th17 cells (35), blockade of the cytokine may bear renoprotective potential in the pre-AKI or initial AKI phases. As increased urinary and, to a lesser extent, serum IL-6 levels predict AKI (71, 78, 79), a test for urinary IL-6 may be considered as an indicator for the administration of IL-6 pathway inhibitors to prevent or alleviate the disease.

## IL-6 signaling in chronic kidney disease

The diagnosis of Chronic Kidney Disease (CKD) relies on one of the following criteria persisting longer than 3 months: reduction of GFR below 60 mL/min/1.73 m^2^, albuminuria of at least 30 mg per 24 hours, or other abnormalities reflecting functional kidney injury or structural kidney damage (hematuria, polycystic or dysplastic kidneys *et cetera*) (89). Affecting over 850 million people worldwide, CKD has been increasingly recognized as a global public health challenge and a major non-communicable human disease (90). CKD frequently develops during progression of the Diabetic Kidney Disease (DKD) (91). Apart from the diabetes mellitus, etiology of CKD comprises various risk factors including unresolved or recurrent AKI, hereditary kidney diseases, congenital anomalies of the urogenital system, autoimmune disorders, hypertension, and obesity (44). Despite various primary causes, progression of CKD is driven by shared pathophysiologic mechanisms associated with tissue hypoxia, oxidative and metabolic stress, chronic inflammation, and fibrosis. Moreover, CKD must be viewed as a systemic disease implicating innate immunity, neuroendocrine control, as well as the cardiovascular, digestive, and respiratory organ systems (92). Maladaptive interactions between kidney and immune cells along with chronic metabolic stress of kidney epithelia are the pathophysiologic hallmarks of CKD progression (60, 68, 69, 93). In this light, dysregulation of IL-6 signaling has been typically observed in CKD patients and animal models. However, etiologic factors substantially affect the diagnostic and prognostic IL-6 significance with the apparently strongest correlation between IL-6 levels and progression to CKD in patients with autoimmune kidney disorders and less clear situation in patients with the Diabetic Kidney Disease (DKD) (35, 94–98).

### Autoimmune kidney diseases

Autoimmune kidney diseases comprise the lupus nephritis (LN), anti-neutrophil cytoplasmic associated (ANCA) vasculitis, anti-glomerular basement disease (also known as Goodpasture’s disease), IgA nephropathy (IgAN), and membranous nephritis (MN) (99). The autoimmunity-mediated renal diseases are caused by loss of self-tolerance to certain own proteins becoming autoantigens and provoking immune-mediated kidney damage, typically manifesting by glomerulonephritis (GN). The autoantigens may be of renal or non-renal origin, the latter accumulate in glomeruli due to the physiologically high renal blood supply and the perm-selective blood filtration. IL-6 has been implicated in pathophysiology of distinct autoimmune GN forms either as a diagnostic marker or a pathogenetic factor (100). Since IL-6 blocking agents are increasingly used to retard progress of inflammatory autoimmune diseases such as rheumatoid arthritis (RA), juvenile idiopathic arthritis, and Castleman’s disease, the amount of clinical information on their effects in patients with autoimmune renal disorders is continuously growing (101, 102).

RA may be complicated by nephropathy basically displaying GN with or without nephrotic syndrome (NS). Notably, renal complications in RA patients may be caused not only by the immune-mediated kidney damage itself but by side effects of antirheumatic drugs as well (103). Although the incidence of RA-associated nephropathy with progression to CKD has substantially declined after the clinical introduction of disease-modifying antirheumatic drugs (DMARDs), renal complications in RA patients remain a problem deserving attention (103). Safety and tolerability of distinct IL-6 signaling inhibitors in RA patients with renal insufficiency have been generally established (104). Nevertheless, systematic studies addressing effects of IL-6 inhibition on the incidence or course of renal complications in RA patients are still scarce and mostly limited to case reports. In this context, several case reports describe beneficial effects of the IL-6R blocker tocilizumab on the renal function in patients with RA-associated GN, mainly due to secondary amyloidosis (105–107). Tocilizumab has further shown nephroprotective effects in patients developing ANCA vasculitis and GN with or without RA in the background (108, 109). The underlying mechanisms are likely related with the systemic anti-inflammatory and immunomodulatory effects of tocilizumab rather than being kidney-specific (110). Since IL-6 has been implicated in the pathophysiology of Systemic Lupus Erythematosus (SLE), effects of IL-6 blockade used to treat the disease have been studied in patients with *Lupus* GN as well. Disappointingly, treatment of SLE patients with an IL-6 inhibitor sirukumab revealed no obvious benefits in those developing *Lupus* GN but was associated with significantly increased adverse effect incidence (111). Finally, IL-6 is believed to promote IgAN (94, 112). Consequently, blockade of IL-6 signaling in patients with IgAN is viewed as an emerging therapeutic option but a supporting clinical evidence is barely available to date. Clinical experience with IL-6 signaling inhibitors in the autoimmune kidney diseases are summarized in the **Table 2A**. Principally, inhibition of IL-6 signaling pathways appears to prevent or retard the autoimmune kidney diseases. The underlying mechanisms mainly rely on general anti-inflammatory and immunomodulatory effects such as the normalization of T cell and macrophage balance by suppressing the pro-inflammatory Th17 and M1, while facilitating the anti-inflammatory Treg and M2 phenotypes (64, 109). The extent of nephroprotection may vary depending on the IL-6 intervention level (IL-6, IL-6R, or JAK/STAT3). Convincing clinical benefits have been currently obtained only for the IL-6R blocker tocilizumab in patients with RA-related or ANCA-associated nephropathy, whereas information on other drugs and indications is still insufficient for evidence-based conclusions.

**Table 2A:** Clinical experience with IL-6 signaling inhibitors in autoimmune kidney diseases.

Disease	Drug	Action	Efficacy	Safety	Reference
RA/GN/NS/AA	Tocilizumab	IL-6R mab	Clinical remission, reduction of proteinuria	well tolerated	Case report (106)
RA/CGN	Tocilizumab	IL-6R mab	Reduction in serum creatinine and proteinuria	well tolerated	Case report (107)
RA, ANCA-GN	Tocilizumab	IL-6R mab	Clinical remission, decreases in RA activity and serum ANCA levels, improved renal findings	well tolerated	Case report (108)
ANCA-GN	Tocilizumab	IL-6R mab	Clinical remission, increase in Treg	well tolerated	Small study (9 AAV patients) (109)
Lupus GN	Sirukumab (CNTO 136)	IL-6, mab	no benefits	↑ total AE rate	NCT01273389 (111)
non-ANCA-associated vasculitis (including IgA)	infliximab, rituximab, and tocilizumab		NA	NA	Phase II, NCT05168475

AA, secondary Amyloid A amyloidosis; AE, Adverse Events; ANCA, Anti-Neutrophil Cytoplasmic Associated; GN, glomerulonephritis; mab, monoclonal antibody; NA, not available; RA, rheumatoid arthritis; ↑, increased.

**Table 2B:** Clinical studies of IL-6 signaling antagonism in diabetic kidney disease.

Disease	Drug	Action	Efficacy	Safety	Reference
T1D	Siltuximab	IL-6 (mab)	not published	NA	NCT02641522, completed (140)
T2D, obesity	Tocilizumab	IL-6R (mab)	not published	NA	NCT01073826, completed (140)
T2D	Tocilizumab	IL-6R (mab)	eGFR stabilization	well tolerated	Case report (141)
T2D	Baricitinib (0.75 mg - 4 mg daily for 24 weeks)	JAK1/JAK2 inhibitor	reduced albuminuria and renal inflammation	well tolerated	NCT01683409 (142)
T2D	Baricitinib (0.75 mg daily; 0.75 mg twice daily; 1.5 mg daily; or 4 mg daily for 24 weeks)	JAK1/JAK2 inhibitor	reduced albuminuria	Increased anemia incidence (highest dose)	NCT01683409, Phase 2 (143)

T1D, type 1 diabetes; T2D, type 2 diabetes; NA, not available.

**Table 2C:** Effects of IL-6 signaling inhibitors in terminal renal insufficiency.

Disease	Drug	Action	Efficacy	Safety	Reference
RA, ESRD	Tocilizumab	IL-6R mab	Good efficacy	Safe, well tolerated	MC study (104)
RA, AA, hemodialysis	Tocilizumab	IL-6R mab	Reduced cardiac hypertrophy	Well tolerated	Case report (149)
AA, pre-dialysis	Tocilizumab	IL-6R mab	Postponed hemodialysis	Well tolerated	Case report (150)
AA, advanced CKD	Tocilizumab	IL-6R mab	Resolution of nephrotic state	Well tolerated	Case report (151)
RA, ESRD, hemodialysis	Tocilizumab	IL-6R mab	Clinical remission	Safe	Case report (152)
RA, AA, ESRD	Tocilizumab	IL-6R mab	↓proteinuria, preservation of GFR, ↓amyloid deposits	Safe	Two case reports (105)
Pharmacokinetics in ESRD	Tofacitinib	JAK1/3 inhibitor		no serious AE	NCT01740362 completed (153)
ESRD on hemodialysis	Tofacitinib	JAK1/3 inhibitor		no serious AE	NCT01710020 completed (153)

AA, secondary Amyloid A amyloidosis; AE, adverse events; GFR, estimated glomerular filtration rate; mab, monoclonal antibody; NA, not available; RA, rheumatoid arthritis; CKD, chronic kidney disease; ESRD, end-stage renal disease; ↓, decreased.

**Table 2D:** Clinical studies of IL-6 signaling antagonism in kidney transplantation.

Indication	Regimen	Mode	Efficacy	Safety	References
Highly HLA-sensitized kidney transplant candidates	Clazakizumab (pre: 25 mg/mo X6 + PLEX, IVIg; post: standard immunosuppression + 25 mg/mo)	IL-6 mab	significant reductions in class I and class II antibodies, no need for further DSA rebound in 18 of 20 patients	well tolerated	(182)
Late AMR kidney transplant rejection	Clazakizumab (25 mg 4/week s.c. for 12 weeks d.b. + 40 weeks open)	IL-6 mab	↓DASs, better biopsy morphology, retarded GFR decline	Risk of serious infections and diverticular disease	(183)
HLA-sensitized kidney transplant cAMR	Clazakizumab (25 mg s.c./mo for 12 mo to 2.5 yy)	IL-6 mab	eGFR stabilization, ↓DSAs, trend for Treg increase	Well tolerated	(177)
cAMR in kidney transplant recipients	Clazakizumab	IL-6 mab	NA yet	NA yet	NCT03744910 (IMAGINE), Phase 3
ESRD awaiting kidney transplantation	Tocilizumab	IL-6R mab	Reduction of donor-specific HLA antibodies, desensitization	well tolerated	NCT01594424, Phase 1/2 (180)
Highly HLA-sensitized kidney transplant candidates	Tocilizumab (8 mg/kg once a mo)	IL-6R mab	Minimal reduction of anti-HLA abs	well tolerated	(189)
Highly HLA-sensitized kidney transplant candidates	Tocilizumab (8 mg/kg once a mo)	IL-6R mab	Minimal effect on anti-HLA abs	well tolerated	(181)
Chronic AMR	Tocilizumab (posttransplant cAMR, DSAs, TG, no response to standard care)	IL-6R (mab)	High rate of graft and patient survival, ↓DSAs, renal function stabilization	well tolerated	(184)
Clinically stable kidney transplant recipients	Tocilizumab	IL-6R mab	Increase in Tregs and reduction in T effector cytokines	well tolerated	NCT02108600, completed (178)
First line cAMR therapy in kidney transplant patients	Tocilizumab	IL-6R mab	GFR and proteinuria stabilization, improved biopsy morphology, regeneration	well tolerated	(186)
Pediatric renal transplant recipients with AMR refractory to IVIg/Rituximab	Tocilizumab (median 12 doses)	IL-6R mab	eGFR stabilization, moderate improvement of morphology	well tolerated, (cases of cytopenia)	(187)
aAMR (on top of standard therapy)	Tocilizumab (8 mg/kg*mo)	IL-6R mab	eGFR improvement or stabilization, ↓DSAs	well tolerated	(185)
cAMR in kidney transplant recipients	Tocilizumab (8 mg/kg*mo)	IL-6R mab	no efficacy	well tolerated	(190)
cAMR in kidney transplant recipients resistant to standard therapy	Tocilizumab (8 mg/kg*mo)	IL-6R mab	no efficacy	well tolerated	(191)
SC retrospective cAMR in kidney transplant recipients	Tocilizumab	IL-6R mab	Clinical and histological benefits	well tolerated	(188)
Promoting tolerance	Recipient Treg cells + Tocilizumab + donor bone marrow	Cell therapy + IL-6R mab	Ongoing	ongoing	NCT03867617
Combined with co-stimulation blockade to maintain Tregs	Tocilizumab + lulizumab for 3 mo -> belatacept + everolimus + prednisolon	IL-6R mab + co-stimulation blockade	not published	not published	NCT04066114
Tofacitinib (CNI-free) vs. Tacrolimus, transplant recipients	Tofacitinib (CP-690,550): 15 vs. 30 mg	JAK1/3 inhibitor	acute rejection rate at 15 mg comparable to tacrolimus	higher rate of viral infections at 30 mg	NCT00106639, NCT00263328, completed (192)
Tofacitinib (CNI-free) vs. Cyclosporine A, transplant recipients	Tofacitinib (CP-690,550)	JAK1/3 inhibitor	comparable allograft survival, higher GFR, less TIN	higher serious infection rate	NCT00483756, NCT00658359, completed (193)
Tofacitinib safety, pharmacokinetics in stable allograft	Tofacitinib (CP-690,550)	JAK1/3 inhibitor		well tolerated	NCT01710033

AMR, antibody-mediated rejection, acute/active AMR (aAMR); chronic AMR, cAMR; DSA, donor-specific antibodies; eGFR, estimated glomerular filtration rate; GN, glomerulonephritis; NA, not available; NS, nephrotic syndrome; RA, rheumatoid arthritis; TG, transplant glomerulonephritis; ↓, decreased.

### Diabetic kidney disease

The Diabetic Kidney Disease (DKD) develops in approximately 40% of individuals with diabetes of either type 1 (T1D) or type 2 (T2D) and is the dominant cause for the Chronic Kidney Disease (CKD) (91, 113). The pathophysiologic mechanisms of diabetic kidney damage combine microvascular injury, glomerular hyperfiltration, toxic effects of hyperglycemia, and associated comorbidities such as hypertension or dyslipidemia (91). The resulting nephron loss and sterile inflammation of renal tissue evoke invasion of immune cells and immune-mediated damage. Proinflammatory cytokines have been principally implicated in cardiometabolic diseases including diabetes (98). In this context, several lines of evidence suggested a pathogenetic role of IL-6 in diabetes and DKD (30).

**Figure 3:**
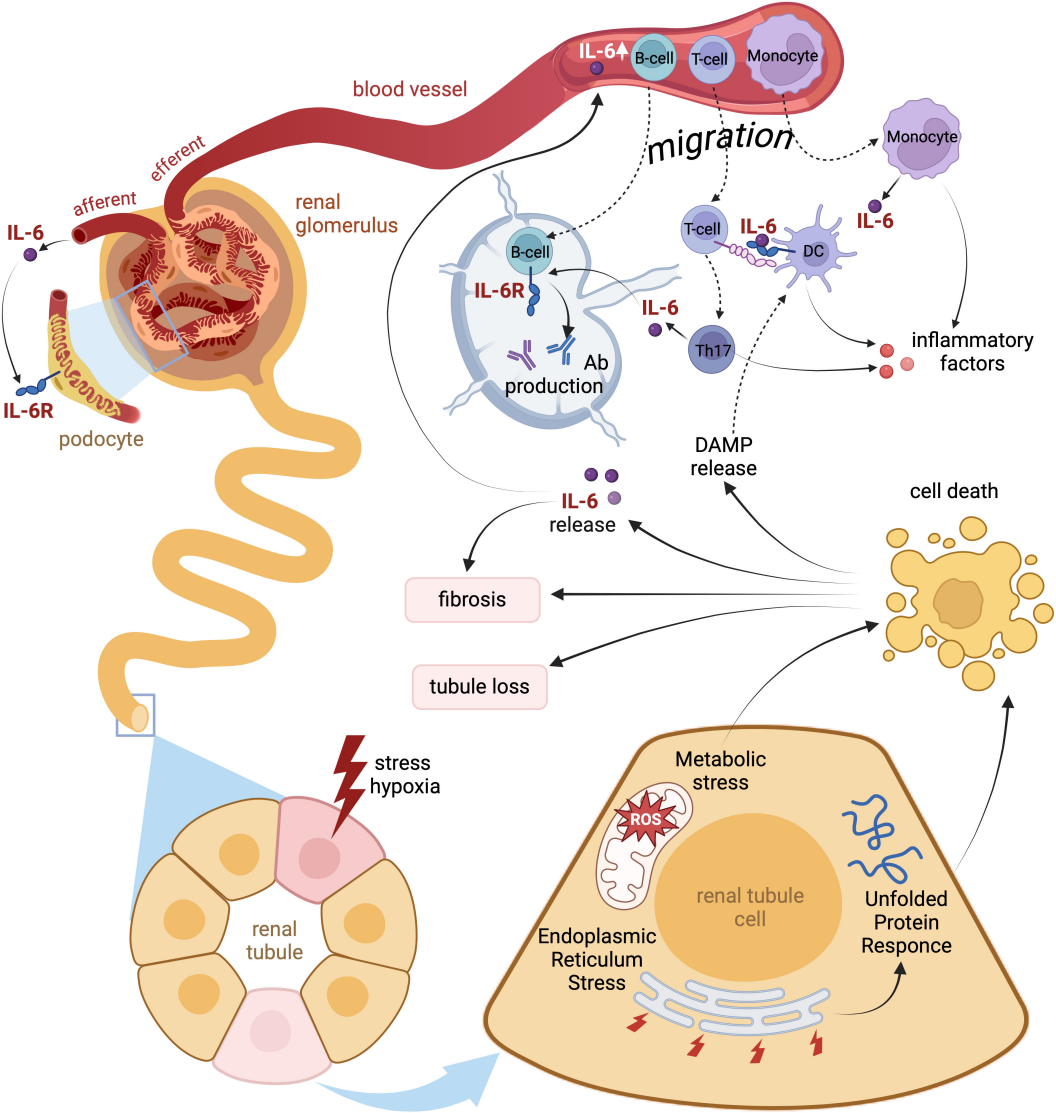
Local and systemic pathophysiologic effects of IL-6 signaling during kidney injury. Acute or chronic hypoxic and metabolic stress of renal tissue breaks intact energy and protein homeostasis in kidney epithelia causing endoplasmic reticulum stress with unfolded protein response. Injured cells signal via enhanced IL-6 secretion thus attracting local immune cells. Decompensation of cell protective adaptations lead to apoptosis of renal tubular cells and release of Damage-Associated Molecular Patterns (DAMP) further enhancing the local immune response. Both IL-6 and DAMP induce invasion of T-cells and monocytes followed by predominant acquisition of pro-inflammatory Th17 and M1 phenotypes. The infiltrating immune cells produce IL-6 and other pro-inflammatory cytokines thus adding to local sterile inflammation of renal tissue. Later migration of antigen-presenting cells exposed to DAMP to lymphatic nodes may trigger production of autoantibodies by B-cells. Finally, the inflamed kidney tissue delivers substantial amounts of IL-6 into the blood thus provoking systemic inflammation.

IL-6 is a cytokine with pleiotropic functions including central and peripheral effects on the glucose homeostasis (114). IL-6 and leptin share signaling mechanisms that suppress feeding and improve glucose tolerance. Central activation of the IL-6 trans-signaling has been reported to improve glucose metabolism in mouse models of obesity (114). Similar to leptin, central IL-6 action may be mediated via activation of oxytocinergic neurons located in in the hypothalamic paraventricular nucleus (115, 116). In contrast to the reported obesity-associated leptin resistance, central IL-6 trans-signaling appears to be stimulated in obesity (114). The hypothalamic neurons may transduce effects of IL-6 on the systemic glucose metabolism by modulation of the sympathetic/parasympathetic tone or via the hypothalamic-pituitary hormonal axis. Apart from that, IL-6-dependent stimulation of insulin secretion may involve gastrointestinal hormones such as the glucagon-like peptide 1 (117). In peripheral tissues, IL-6 promotes insulin-dependent glucose utilization in skeletal muscles along with lipolysis in the fat tissue (118–120). In the liver, however, the cytokine induces insulin resistance reflected by blunted synthesis but enhanced degradation of glycogen, as well as facilitated gluconeogenesis (121). All these effects of the cytokine are part of catabolic metabolism serving to improve energy mobilization in response to challenge. The physiologic “antidiabetic” effects of the cytokine need to be taken into consideration for understanding its role in diabetes and DKD. Nevertheless, prolonged stimulation of the IL-6 signaling during chronic systemic inflammation coincides with development of insulin resistance (122). Moderately enhanced plasma IL-6 levels have been frequently reported in T2D patients but the pathophysiological meaning of this finding remains debatable (123–125). Thorough matching the T2D patients with the respective control patients for age, weight, sex, and BMI revealed no significant differences in plasma IL-6 levels suggesting that the fat mass but not impaired insulin responsiveness underlies the elevated IL-6 plasma levels in diabetic patients (126). Indeed, IL-6 mRNA expression was demonstrated in human subcutaneous adipose tissue and elevated IL-6 mRNA levels measured in individuals with insulin resistance (127, 128). While molecular pathways connecting IL-6 to insulin resistance in the adipose tissue remain elusive, stimulation of IL-6 synthesis may reflect a compensatory response to impaired glucose metabolism (122). Therefore, the fat tissue constitutes a large source of circulating IL-6 in patients with diabetes and metabolic syndrome independently on the kidney involvement (124). DKD adds the kidney as another major site of IL-6 production, since the extent of kidney damage appears to correlate with increased renal IL-6 mRNA expression in DKD patients (97). Furthermore, enhanced circulating or urinary IL-6 levels have been associated with the risk of kidney disease progression in T1D and T2D patients (96, 129, 130). The underlying pathophysiological mechanisms are strongly related to the immune-mediated kidney damage induced by infiltrating Th17 lymphocytes and M1 macrophages (64, 67–69). The invasion of immune cells might be provoked by IL-6 release from injured kidney cells, followed by mutual paracrine stimulation of proinflammatory cytokine release promoting kidney damage and fibrosis. Once initiated, this vicious pathogenetic circle may become largely independent on circulating IL-6 levels due to amplified local IL-6 synthesis and release in the kidney tissue (**Figure 3**). Notably, monocyte-derived macrophages from newly diagnosed untreated T2D patients showed upregulated activity of the *nucleotide binding and oligomerization domain-like receptor family pyrin domain-containing 3* (NLRP3)-inflammasomes along with the stimulated proinflammatory cytokine expression profile (131). Apart from immune cells, activation of NLRP3-inflammasomes has been reported in kidney epithelial and endothelial cells from diabetic humans and mice. Moreover, experimental evidence strongly implicates activation of NLRP3-inflammasome in non-immune kidney cells upon DKD (132). NLRP3-inflammasomes lead to maturation of IL-1β and IL-18, whereas IL-6 is a known downstream target of IL-1β with potential synergistic proinflammatory effects (133, 134). In line with this, the IL-6R inhibitor tocilizumab retarded DKD in a mouse model of obesity- and diabetes-induced DKD (db/db mice) mainly via suppressed activation of NLRP3-inflammasomes and blunted immune-mediated kidney damage (135). In contrast, genetic IL-6 deletion in mice provided no protection against the obesity-induced renal impairment but aggravated nephrotoxic effects of high fat diet-induced instead (136). Notably, IL-6 knockout mice develop mature-onset obesity implicating IL-6 as an essential player in the carbohydrate and lipid metabolism (137). Accordingly, administration of IL-6 pathway inhibitors to treat autoimmune diseases was associated with increased body weight and body mass index (BMI) in humans (138). At the same time, IL-6R blockade has been shown to improve insulin sensitivity in non-diabetic patients receiving tocilizumab for management of rheumatoid arthritis (139). The aforementioned discrepancies may reflect a disbalance between the central and peripheral IL-6 signaling in diabetes. With respect to the relevant IL-6 signaling modes, data from DKD patients, mouse models, and cell culture involve both the classic and the trans IL-6 signaling in diabetic kidney injury (13, 123). Further dissection between central and peripheral effects of the cytokine and distinct signaling modes is mandatory to carefully assess the therapeutic potential of IL-6 inhibiting agents in diabetes and DKD.

Despite growing number of animal studies suggesting beneficial effects of IL-6 suppression in DKD and CKD, clinical experience with inhibitors of IL-6 pathways in diabetic patients are rather limited and less conclusive (**Table 2B**). Two kidney-focused clinical trials testing either suppression of IL-6 with siltuximab (NCT02641522) in T1D patients or inhibition of IL-6R with tocilizumab in patients suffering from T2D and obesity (NCT01073826) were conducted but the results and conclusions are pending (140). A case report documented rescue of the renal function in a patient with DKD receiving tocilizumab (141). Administration of baricitinib in T2D patients to suppress JAK downstream of IL-6R showed acceptable safety and a moderate improvement of the kidney function reflected by reduced albuminuria (142, 143). While potential benefits of systemic IL-6 inhibition in DKD patients require further investigation, selective renal targeting of the IL-6 signaling to suppress immune cell invasion and local maturation of Th17 cells and M1 macrophages may open a new therapeutic avenue for improved management of DKD and CKD.

### End-stage renal disease

ESRD is a terminal step of kidney insufficiency with GFR below 15 mL/min, which is a life-threatening condition requiring Renal Replacement Therapy (RRT) in form of hemodialysis, peritoneal dialysis, or kidney transplantation. The main causes of ESRD in developed countries comprises DKD, hypertension, primary and secondary systemic vasculitis, polycystic kidney disease, obstructive nephropathy or vesicoureteral reflux, renal amyloidosis, and drug nephrotoxicity (144). Irreversible kidney injury in ESRD is associated with systemic inflammation provoking multiple dysfunctions of internal organs, skeletal muscles, and integumentary tissues with risk of ensuing cardiorenal syndrome, hepatorenal syndrome, respiratory disorders, cerebrovascular pathology, muscle atrophy, and cahexia (145). Proinflammatory cytokines including IL-6 have been implicated in progression of CKD to ESRD (129). Circulating IL-6 levels are markedly increased in ESRD patients, both newly diagnosed or receiving hemodialysis (146). The reported highest IL-6 concentrations in patients with ESRD ranged within 60-150 pg/ml thus manyfold exceeding moderately increased IL-6 levels in DKD (mostly below 10 pg/ml) (130, 146). Persisting elevation of plasma IL-6 levels has been proven as a hallmark of CKD aggravation and a predictor of overall and cardiovascular mortality in pre-dialysis and hemodialysis CKD patients (147). Notably IL-6 emerges as a more reliable prognostic marker of CKD outcomes than the C-reactive protein, albumin, or tumor necrosis factor (147). Since ESRD provokes systemic inflammation, the resulting injury of other organs and tissues may provide multiple sources for enhanced circulating IL-6 levels in addition to the damaged kidneys. Pharmacologic suppression of the IL-6 signaling to attenuate systemic inflammation and the CKD/ERSD-associated cardiovascular burden appears rational in this setting (148). Although the clinical experience with IL-6 inhibitors in ESRD patients is scarce and mostly limited to the IL-6R blocker tocilizumab, an acceptable safety level and beneficial effects on the renal and cardiovascular outcomes were consistently reported in pre-dialysis or hemodialysis patients (105, 149–152). Administration of the JAK-inhibitor tofacitinib to patients with mild to severe renal insufficiency demonstrated satisfactory pharmacokinetics suggesting that tofacitinib may be used in ESRD patients as well (**Table 2C**) (153).

## Kidney transplantation

Kidney transplantation represents the most vulnerable condition for the renal injury. The transplanted kidney subjected to the ischemia–reperfusion injury reacts to hypoxic and metabolic stress by agile IL-6 production, thereby eliciting or aggravating own rejection via cellular and humoral alloimmune responses (154). Enhanced urinary IL-6 or sIL-6R levels have been increasingly recognized as early diagnostic markers of graft rejection associated with poor prognosis (3, 83, 155–160). The transplanted kidney appears to be the main source of increased circulating and urinary IL-6 originating primarily from tubular epithelial cells, monocytes and macrophages (161, 162). The impact of kidney-derived IL-6 on the graft survival has been studied in experimental transplantation models using wild-type (WT) and IL-6-deficient mice (163). The serum IL-6 levels in mice receiving WT kidney transplantation were substantially higher compared to IL-6-deficient kidney recipients, the latter were even comparable to the levels detected in sham-operated mice. Accordingly, grafts from IL-6 knockout mice demonstrated prolonged rejection-free time suggesting major contribution of graft-derived IL-6 to the development of transplant rejection (163). Furthermore, IL-6 interfered with Treg activity against the effector T cell responses and impaired graft acceptance in non-renal murine transplant models (164, 165).

Clinically, increased urinary or serum IL-6 levels coincide with inflammation, acute rejection, and chronic rejection of renal allografts (155–157). High serum IL-6 levels have been strongly associated with ensuing transplant rejection among renal allograft recipients undergoing tolerance induction using a mixed chimerism strategy (166). Moreover, increased urinary IL-6 levels accompany delayed graft function and resolve in a tight correlation with functional improvement of the transplant (158). The underlying mechanisms are complex and include stimulation of the pro-inflammatory Th17 cells, suppression of the anti-inflammatory Treg cells, enhanced production of alloantibodies, as well as local pro-inflammatory tissue reactions (154).

A growing body of experimental and clinical evidence points to significant therapeutic potential of IL-6 signaling inhibitors in prevention and treatment of acute and chronic renal graft rejection (3). Phenotyping of IL-6 knockout mice revealed a highly favorable T cell profile with the strongly reduced to absent Th17 response by the dominating FoxP3^+^ Treg phenotype promoting immune tolerance (167). Although the differentiation of naïve T cells into Th17 does not solely depend on IL-6 and can be alternatively achieved by combined effects of IL-21, tumor growth factor beta (TGF-β) and IL-23 (168–171), sustained IL-6 signaling plays an essential role in maintaining Th17 cell identity while suppressing Treg cells (172). Blockade of IL-6R using tocilizumab to treat RA led to sustained increase in circulating Treg levels during the therapy suggesting that IL-6 inhibition may improve the Th17/Treg balance in kidney transplantation as well (173, 174). In contrast to Treg cells, tocilizumab-induced changes in the percentage of Th17 cells were less conclusive. Cytokines and factors other than IL-6 may help maintain the Th17 response, as described above (167, 175). However, suppression of the IL-6 signaling retards the acquisition of Th17 pathogenic phenotype (176). Effects of IL-6 inhibition on the Th17/Treg balance in the setting of kidney transplantation have not been systematically investigated so far, although the available data suggests favorable effects (177, 178). Moreover, increased circulating Treg levels and clinical benefits were observed in patients receiving IL-6R blockade by tocilizumab for management of autoimmune disorders at risk or during manifestation of autoimmunity-mediated renal disease (106, 107, 109). Ongoing studies on therapeutic potential of the IL-6R inhibitor tocilizumab to promote immunologic tolerance in combination with recipient Treg cells (NCT03867617) or to rescue Treg functionality during co-stimulation blockade with belatacept (NCT04066114) may translate the experimental findings to the clinic (179).

The current clinical experience with IL-6 signaling inhibitors in kidney transplant recipients has been primarily derived from tocilizumab and clazakizumab employed as a strategy to manage the humoral alloimmune response, i.e. either for human leukocyte antigen (HLA) desensitization before transplantation or treatment of chronic antibody-mediated rejection (AMR) after the transplantation (177, 180–182). In general, IL-6 inhibition using tocilizumab or clazakizumab was associated with reduction of donor-specific antibody titers (DSA, anti-HLA) (177, 180, 182–185), stabilization or improvement of kidney function (177, 183, 184, 186, 187), reduction of proteinuria (186), and benefits for kidney morphology (183, 186–188). Notably, reduction in eGFR correlated with decrease in DSA titers and resolution of histological abnormalities in patients receiving clazakizumab suggesting that benefits of IL-6 inhibition are significantly mediated by suppression of B cell activity (183). Despite noticeable number of side effects, mainly infections and diverticulitis, clazakizumab was generally well tolerated by kidney transplant recipients receiving concomitant immunosuppression (177, 182, 183). The efficacy of tocilizumab appears less conclusive according to several reports (181, 189–191). However, no direct comparison between the two drugs was conducted so far and the question of preferential IL-6 vs. IL-6R targeting for better graft outcomes remains open.

Further clinical experience was obtained with JAK/STAT3 inhibitors such as tofacitinib acting downstream of IL-6R (153, 192, 193). Notably, tofacitinib provided sufficient immunosuppression levels as monotherapy, comparable to the efficacy of calcineurin inhibitors being currently the first-line immunosuppressive regiment in transplant recipients worldwide. At the same time, use of tofacitinib was associated with higher general and serious infection rate requiring further studies and protocol adjustmens (193). In any case, the observed strong immunosuppressive efficacy of tofacitinib suggests similar therapeutic potential of IL-6 or IL-6R inhibitors in partial or complete substitution of highly nephrotoxic calcineurin inhibitors (194).

The available clinical experience with IL-6 signaling inhibitors in kidney transplantation is summarized in the **Table 2D**. Since the kidney is the most frequently transplanted organ, data on targeting the IL-6 signaling in non-renal transplant recipients are still limited but could be carefully interpreted as corroborating the therapeutic potential of IL-6 inhibition as well (NCT03644667 ongoing) (195, 196).

## Choice of proper IL-6 signaling inhibitors in kidney diseases

IL-6 induction affects pathophysiology of major non-infectious kidney disorders including renal complication of autoimmune diseases, AKI, CKD, and rejection of renal allografts. Accumulating clinical experience points to therapeutic potential of IL-6 suppression in renal patients but diagnostic criteria and treatment protocols for administration of different IL-6 signaling inhibitor types need to be established. Complete or partial silencing of IL-6 signaling can be principally achieved by inhibition of IL-6, IL-6R, gp130, or the downstream JAK/STAT3 pathway (1). While IL-6 expression is strongly induced during inflammation, parallel changes in the mIL-6R or mgp130 expression levels are rather mild. Use of IL-6-neutralizing antibodies appears plausible in this context (10). In contrast, initially high doses of antibodies targeting IL-6R would be required for saturating inhibition of its membrane-bound and soluble variants. Finally, pharmacologic suppression of gp130 is not reasonable since many cytokines share gp130 for signaling events and gp130-knockout mice exhibit multiorgan pathology (197). Similarly, small-molecule inhibitors of JAKs target not only IL-6 but other cytokines recruiting the JAK/STAT3 pathway (1, 10). Based on these considerations, flexible dosage adjustment of IL-6-neutralizing antibodies may represent a viable approach towards a personalized therapeutic effect in various kidney diseases.

Selective suppression of distinct IL-6 signaling pathways may provide further therapeutic benefits. Among the three IL-6 signaling routes, the trans-signaling has been generally established as the major mediator of pro-inflammatory and pathophysiologic effects. Identification of sgp130 as an endogenous trans-signaling inhibitor forced development of recombinant sgp130 variants for therapeutic purposes (10). The first generated sgp130 variant olamkicept comprises six extracellular domains of gp130 fused to an Fc-part of an IgG antibody (sgp130Fc) and is intended for selective suppression of the IL-6 trans-signaling pathway, while permitting the classic mode. Clinical testing of olamkicept as an anti-inflammatory and immunosuppressive agent started in 2012 and delivered promising results so far (10, 198, 199). A certain cross reactivity of olamkicept with the IL-11 trans signaling was resolved by later refinements in drug design resulting in sgp130 variants with predominant to exclusive selectivity for IL-6 over IL-11 (200). Therefore, olamkicept and its next generation drugs may efficiently buffer the trans-signaling events in kidney diseases as well. Moreover, sgp130 analogues have been shown to suppress the cluster IL-6 signaling along with the trans-signaling pathway (15). Although the pathophysiological impact of the cluster (trans-presentation) signaling in kidney diseases has not been clearly established, it is tempting to speculate that this signaling mode may promote the priming of Th17 cells by the renal dendritic cells (16). Notably, due to intracellular preformation of IL-6:IL-6R complexes by presenting cells, the cluster signaling appears to be resistant to IL-6 neutralizing antibodies, with a probable exception of olokizumab. Olokizumab blocks the functional hexamer assembly by occupying the relevant binding site in IL-6 (site III) thereby being able to suppress the signaling even after successful presentation of the IL-6:IL-6R complex to a mgp130-expressing cell. The ability of olokizumab to inhibit the cluster-signaling needs further experimental verification to explore its potential as a pan-inhibitor of all IL-6 signaling modes.

Despite promising therapeutic potential of IL-6 inhibiting drugs in kidney diseases, the translation to the nephrological field is delayed by the relative scarcity of clinical information from renal patients and the absence of clear guidelines, respectively. Nevertheless, IL-6 inhibiting agents have been increasingly implemented for prevention and treatment of kidney transplant rejection and retardation of autoimmune kidney diseases. The great majority of clinical data was obtained using the IL-6R neutralizing antibody tocilizumab (105, 188). Nevertheless, we believe that available monoclonal antibodies to IL-6 bear comparable or even superior therapeutic potential. More clinical studies are mandatory to establish further IL-6 inhibiting agents to combat the acute and chronic allo- and autoimmune reactions. Use of new agents selectively suppressing trans-signaling, such as olamkicept, needs further characterization due to expected clinical benefits. Apart from the kidney transplantation setting, ESRD would obviously profit from IL-6 inhibitors as well. Since ESRD leads to systemic inflammation and multiorgan damage, suppression of IL-6 signaling is justified (145). Any type of IL-6 inhibiting agents would potentially be applicable but further clinical investigation is needed to our opinion. More complex is the situation with DKD, since IL-6 may have certain “antidiabetic” effects (114). Furthermore, podocytes may profit from the classic IL-6 signaling but the trans-signaling aggravates glomerular inflammation (201). Therefore, selective suppression of the trans-signaling using olamkicept may be considered as the primary choice but this speculation requires experimental support. Along the same line, olokizumab may provide benefits due to its putative ability to suppress the cluster-signaling, which may reduce the immune-mediated kidney damage. However, effects of distinct IL-6 inhibiting agents including olamkicept and olokizumab in DKD require further detailed investigation. The same applies to AKI, the understanding of the cytokine effects in the AKI pathophysiology including the injury and repair phases need to be improved and different IL-6 inhibitors tested.

Taken together, IL-6 has been increasingly recognized as a major player in kidney pathophysiology due to its role in sterile inflammation and immune-mediated damage. IL-6 inhibiting therapy has entered the nephrological care with clinically proven benefits for renal transplant recipients and emerging perspective in a wide range of kidney diseases. In addition, IL-6 inhibitors are expected to reduce risk of inflammation-associated cardio-vascular complications of kidney diseases. Therefore, introduction of IL-6 signaling inhibitors into clinical nephrology bears great potential to reduce the morbidity and mortality related to kidney disease. Improved understanding of the interplay between different IL-6 signaling modes in kidney diseases along with accumulation of clinical experience with IL-6 inhibiting drugs in renal patients will define concrete therapeutic avenues.
